# Synchronous and metachronous liver metastases in patients with colorectal cancer—towards a clinically relevant definition

**DOI:** 10.1186/s12957-019-1771-9

**Published:** 2019-12-26

**Authors:** Jennie Engstrand, Cecilia Strömberg, Henrik Nilsson, Jacob Freedman, Eduard Jonas

**Affiliations:** 1 Division of Surgery, Department of Clinical Sciences, Karolinska Institutet, Danderyd Hospital, 182 88 Stockholm, Sweden; 2 Division of Surgery, Department of Clinical Science, Intervention and Technology (CLINTEC), Karolinska Institutet, Karolinska University Hospital, Stockholm, Sweden; 3 Surgical Gastroenterology Unit, Department of Surgery, Groote Schuur Hospital, University of Cape Town Health Sciences Faculty, Cape Town, South Africa

**Keywords:** Colorectal cancer, Liver metastases, Synchronous, Metachronous

## Abstract

**Background:**

Approximately 25% of patients with colorectal cancer (CRC) will have liver metastases classified as synchronous or metachronous. There is no consensus on the defining time point for synchronous/metachronous, and the prognostic implications thereof remain unclear. The aim of the study was to assess the prognostic value of differential detection at various defining time points in a population-based patient cohort and conduct a literature review of the topic.

**Methods:**

All patients diagnosed with CRC in the counties of Stockholm and Gotland, Sweden, during 2008 were included in the study and followed for 5 years or until death to identify patients diagnosed with liver metastases. Patients with liver metastases were followed from time of diagnosis of liver metastases for at least 5 years or until death. Different time points defining synchronous/metachronous detection, as reported in the literature and identified in a literature search of databases (PubMed, Embase, Cochrane library), were applied to the cohort, and overall survival was calculated using Kaplan-Meier curves and compared with log-rank test. The influence of synchronously or metachronously detected liver metastases on disease-free and overall survival as reported in articles forthcoming from the literature search was also assessed.

**Results:**

Liver metastases were diagnosed in 272/1026 patients with CRC (26.5%). No statistically significant difference in overall survival for synchronous vs. metachronous detection at any of the defining time points (CRC diagnosis/surgery and 3, 6 and 12 months post-diagnosis/surgery) was demonstrated for operated or non-operated patients. In the literature search, 41 publications met the inclusion criteria. No clear pattern emerged regarding the prognostic significance of synchronous vs. metachronous detection.

**Conclusion:**

Synchronous vs. metachronous detection of CRC liver metastases lacks prognostic value. Using primary tumour diagnosis/operation as standardized cut-off point to define synchronous/metachronous detection is semantically correct. In synchronous detection, it defines a clinically relevant group of patients where individualized multimodality treatment protocols will apply.

## Background

In the USA as well as in Europe, colorectal cancer (CRC) is the third most common cancer and a leading cause of cancer-related death [1]. Approximately 25% of patients diagnosed with CRC will be diagnosed with liver metastases during the course of the disease [2–4]. A large number of clinic-pathologic features of CRC liver metastases (CRCLM), including patient characteristics, pre-operative factors, primary tumour and liver metastases characteristics and operative factors have been assessed as prognostic factors [5, 6]. Synchronous vs. metachronous detection or occurrence of CRCLM as prognostic factor was mainly investigated and reported in surgical case series [7, 8]. There is, however, no consensus on the definition of synchronous and metachronous as used in the context of CRCLM. The time point of diagnosis of the primary tumour, alternatively the time of operation of the primary tumour and a variation of time intervals related to these time points have been used [3]. An effort to solve this was made by Adam et al. in 2015, where synchronous was defined as liver metastases detected before or at the time of diagnosis of the primary tumour [9]. With current trends and developments in CRC and CRCLM treatment, the detection of liver metastases at the time of diagnosis of the primary tumour has important therapeutic implications, both in terms of surgical strategy and the planning of oncologic treatment [10–13]. In a previously published population-based cohort of patients with CRC, the time of detection of liver metastases (synchronous vs. metachronous) did not significantly influence survival in a multivariate analysis [4]. In this study, previously used definitions of synchronous vs. metachronous detection of CRCLM are applied on that same patient cohort to assess the prognostic value of detection at the various defining time points in a population-based cohort. To identify previously used definitions for synchronous vs. metachronous detection of CRCLM, a literature search was performed for articles where a time point was specified for synchronous vs. metachronous detection.

## Methods

To assess the potential prognostic impact of detection of liver metastases at various time points used to define synchronous vs. metachronous detection of CRCLM, a population-based patient cohort was used. All patients that were diagnosed with CRC in the Swedish counties of Stockholm and Gotland between 1 January 2008 and 31 December 2008 (total population as of 1 November 2008 = 2,034,886) were identified in the Swedish Colorectal Cancer Registry (SCRCR) and included in the study. The SCRCR is a validated database covering more than 99% of colon cancers diagnosed in Sweden between 2007 and 2011 [14]. Data regarding metastatic disease is not registered in the SCRCR and in order to identify patients in whom liver metastases were detected, the electronic patient records of all included in the study were reviewed for at least 5 years after time of diagnosis of the primary tumour, or until time of death. The authors reviewed all imaging findings and notifications of intra-operative detection of metastases and documented the occurrence of any metastatic disease. Specifically, the time points for diagnosis of the primary tumour, surgery for the primary tumour, diagnosis of liver metastases and in deceased patients’ time of death were documented. A detailed description of the data collection and demographic and clinic-pathological features of all included patients has been published elsewhere [4]. In that paper, a slightly different definition of synchronous vs. metachronous was used with some liver metastases detected after treatment allocation but during neoadjuvant chemotherapy were categorized as synchronous. The overall survival (OS) of patients with synchronously vs. metachronously detected liver metastases was compared for different time points defining synchronous vs. metachronous detection, identified from the literature search as described below. The calculation was performed for the group of patients with liver metastases as a whole, as well as separately for patients undergoing curative-intended liver intervention (resection and/or local ablation) and those treated with palliative intent. OS was estimated from time of diagnosis of liver metastasis to death, last follow-up or censored January 21, 2019.

In order to define previously used time point definitions of synchronous vs. metachronous detection, a literature search was performed for publications between 2005 and 2018 that described definitions of synchronous vs. metachronous detection. The reported prognostic value of the distinction in articles where OS and/or disease-free survival (DFS) were reported was also documented. The PubMed, Embase and the Cochrane library databases were searched, and articles were reviewed following the PRISMA statement guidelines [15]. The following search headings were used: (CRC OR colorectal cancer) AND (liver OR hepatic) AND (metastases OR metastasis OR metastatic) AND (synchronous OR metachronous) AND survival. Articles not written in English, duplicates, conference abstracts, case reports, review articles and articles written before 2005 were excluded. The remaining articles were subjected to a more thorough screening. For inclusion, the studies had to (a) specify a definition of synchronous vs. metachronous detection of CRCLM and (b) assess synchronous vs. metachronous detection as a prognostic factor (as OS and/or DSF). If the same population was used in two different studies, the later study was included. Publications not meeting the inclusion criteria were deemed irrelevant. A reference screening was performed to detect possible missed articles. The study was approved by the Regional Ethical Review Board in Stockholm.

## Statistical analysis

Non-normally distributed continuous data are presented as medians (min, max) and categorical data as frequencies (percentage). Survival curves were calculated using Kaplan-Meier estimates, and survivor functions were compared using the log-rank test. The threshold for statistical significance was set to *a* < 0.05. Sigmaplot 13 (Systat Software, San Jose, CA 95131, USA) were used for the statistical analyses.

## Results

### Survival data from the population-based cohort

**Fig. 1:**
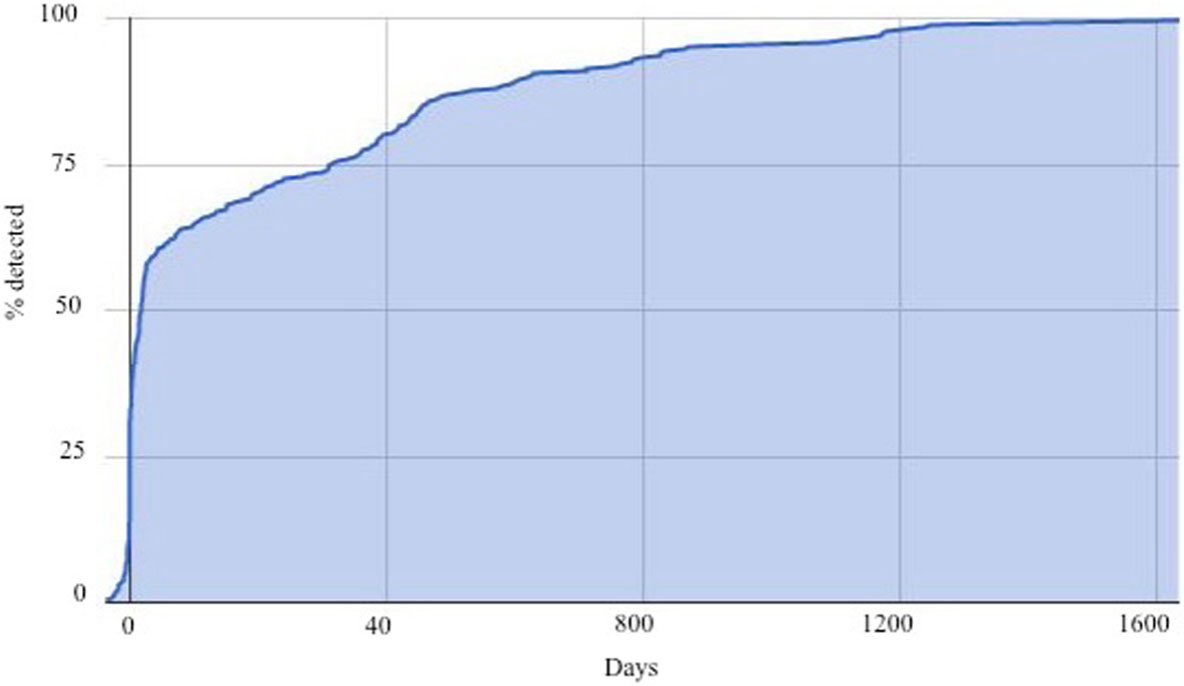
The time of detection of the liver metastases as related to the time of detection of the primary tumour (non-operated, palliative)/operation for the primary tumour (resected)

During the study period, a total of 1026 patients were diagnosed with CRC. The median age was 71 years (31, 97), 485 (47.3%) were females and 651 (65.1%) had a primary tumour of left-sided origin. At 5-year follow-up, 272 (26.5%) of subjects (57.7% male and 42.3% female) had been diagnosed with liver metastases of whom 65 (24%) had undergone liver resection, Table 1. The cumulative incidence of detection of the liver metastases as related to the time of diagnosis of the primary tumour are depicted in Fig. 1. The number of patients with metastases that were classified as synchronous or metachronous according to the different time points used in the literature, namely the time of primary tumour diagnosis (in non-operated patients) or time of primary tumour surgery (operated patients) and 3, 6 and 12 months after the primary tumour diagnosis/surgery, is shown in Table 2.

**Table 1:** Patient and tumour characteristics of 272 patients with liver metastases

	*n* = 272
Gender, male/female (%)	157/115 (57.7/42.3)
Age, years (min, max)	68 (31, 95)
Primary tumour location, *n* (%)	
Caecum/ascending colon	64 (23.5)
Transverse colon	13 (4.8)
Descending/sigmoid colon	90 (33.1)
Rectum	95 (34.9)
Multiple primary tumours	9 (3.3)
Unknown	1 (0.4)
Nodal stage of primary, *n* (%)	
N0	43 (15.8)
N1	141 (51.8)
N2	37 (13.6)
Unknown	51 (18.8)
Tumour stage of primary, *n* (%)	
T0	1 (0.4)
T1	5 (1.8)
T2	7 (2.6)
T3	131 (48.2)
T4	96 (35.3)
Unknown	32 (11.7)
No. of liver metastases, *n* (%)	
1	55 (20.2)
2–5	96 (35.3)
6–10	34 (12.5)
**≥** 11	87 (32.0)
No. of involved segments	
1–2	111 (40.8)
3–4	45 (16.6)
5–6	36 (13.2)
7–8	80 (29.4)
Size of largest liver metastasis (mm) (min, max)	26 (5, 120)

**Table 2:** The number of patients with liver metastases classified as synchronous vs. metachronous according to the different defining time points.

Time point	Synchronous			Metachronous		
	Total	Resected	Non-resected	Total	Resected	Non-resected
Primary tumour diagnosis/surgery	155	26	129	117	39	78
Three months post-primary tumour diagnosis/surgery	174	29	145	98	36	62
Six months post-primary tumour diagnosis/surgery	186	34	152	86	31	55
Twelve months post-primary tumour diagnosis/surgery	207	39	168	65	26	39

**Fig. 2:**
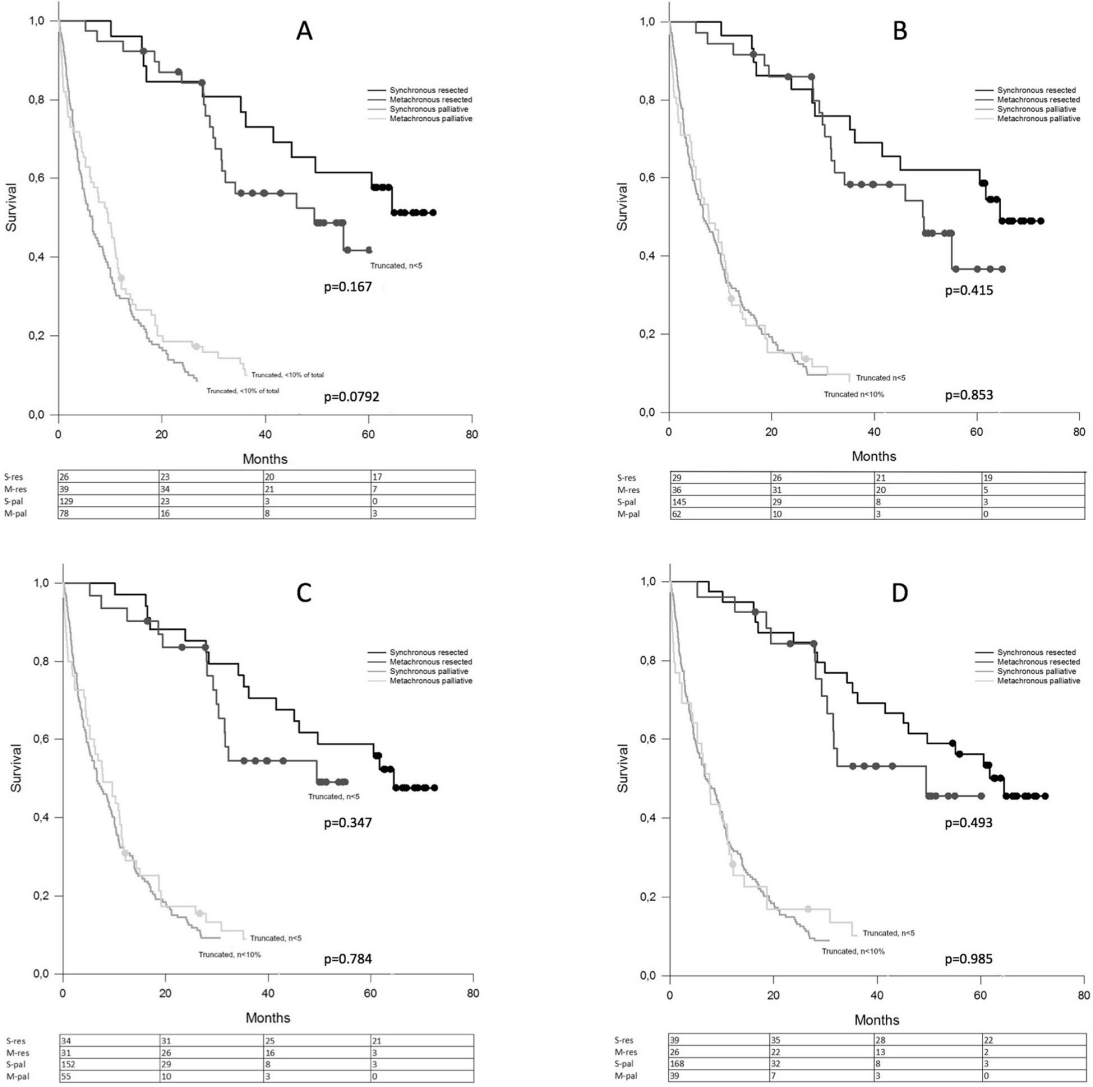
**a**–**d** Overall survival curves for synchronous and metachronous detected metastases. Operated and non-operated patients shown for the different synchronous/metachronous cut-off points at **a**, detection of the primary tumour (non-operated, palliative)/operation for the primary tumour (resected) and **b**, 3 months, **c**, 6 months and **d**, 12 months after detection/resection of the primary tumour

The overall survival curves of patients with synchronously and metachronously detected metastases for operated and non-operated patients are shown for the different synchronous vs. metachronous-defining time points (Fig. 2). No statistically significant difference in survival at any of the time points was seen, neither for operated nor for non-operated patients.

### Review of existing literature on the topic

**Fig. 3:**
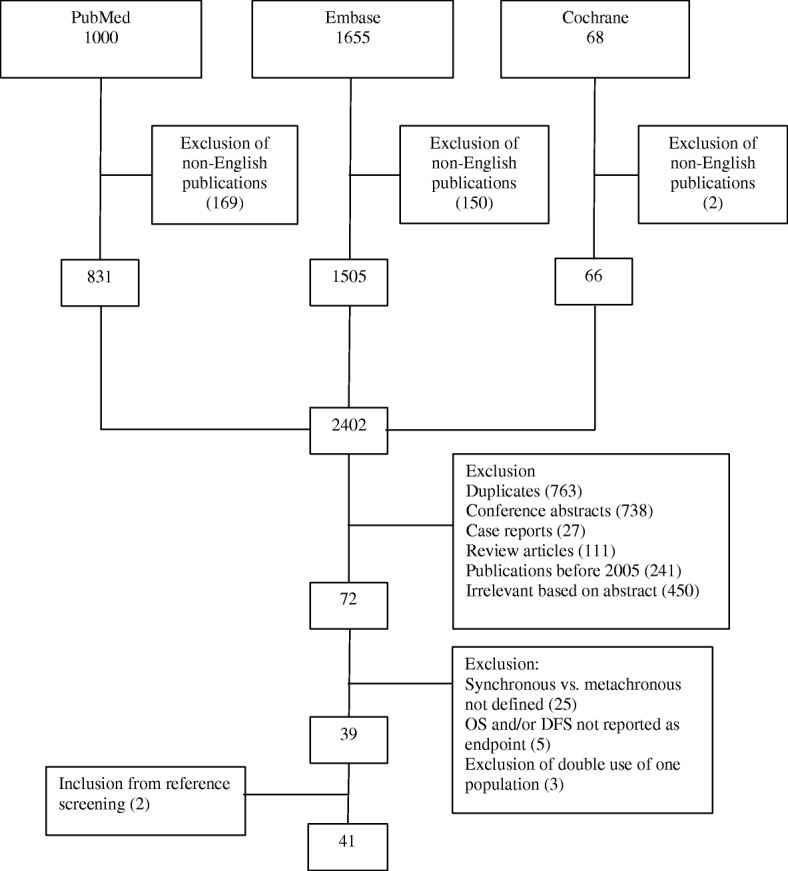
The flow chart presenting the results of the electronic database search

The flow chart presenting the results of the electronic database search is shown in Fig. 3. After exclusion, 39 articles were retained, and 2 articles were added from reference screening. The majority of studies reported patients only operated for CRCLM (*n* = 34), 6 included both operated and non-operated patients and 1 study only included patients treated with palliative intent (Table 3). Prognostic results as per time point are summarized in Table 4. There was a considerable variation in the number of subjects included in studies (minimum 40, maximum 1784), and the proportion of patients with synchronous detection of liver metastases varied from 31.3 to 79.7%. The most commonly used defining time point for synchronicity was at primary tumour diagnosis/surgery (18 out of 41 publications). There was a trend in a better OS of metachronous detected metastases with an earlier cut-off with 50% of studies showing statistically significant differences when using a 0- or 3-month definition, compared to 33% with a 6-month definition and 0% for a 12-month definition; the majority of the studies concluded a non-significant prognostic value of synchronous vs. metachronous detection.

**Table 3:** Studies comparing the prognostic value of synchronous vs. metachronous detection of CRCLM published in 2005–2018

Reference	Year of publication	Cohort	Definition*	Synchronous/metachronous	OS	DFS
Mutsaerts et al. [16]	2005	Operated	2	43/59	S (*p* = 0.048)	NR
Leporrier et al. [17]	2006	All	3	250/108	NS (*p* = 0.498)	NR
Minagawa et al. [18]	2006	Operated	1	187/182	NS (*p* = 0.19)	NR
Taniai et al. [19]	2006	Operated	4	67/41	NS (*p* = 0.354)	NR
Shimizu et al. [20]	2007	Operated	3	70/94	NS (*p* = 0.738)	NR
Tsai et al. [21]	2007	Operated	1	97/58	NS (*p* = 0.150)	S (*p* = 0.004)
Wang et al. [22]	2007	Operated	3	514/409	NS (*p* = 0.312)	NR
Bockhorn et al. [23]	2008	Operated	4	101/101	NS (*p* = 0.78)	NS (*p* = 0.28)
Hamady et al. [24]	2008	Operated	4	138/46	NS (*p* = 0.6)	NR
Vigano et al. [25]	2008	Operated	1	55/66	S (*p* = 0.011)	NR
Konopke et al. [26]	2009	Operated	1	70/131	S (*p* = 0.030)	NR
Ng et al. [27]	2009	Operated	2	35/20	NS (*p* = 0.075)	NS (*p* = 0.43)
Xu et al. [28]	2009	Operated	3	379/290	NS (*p* > 0.05)	NR
Tonelli et al. [29]	2010	Operated	1	70/37	S (*p* = 0.018)	NR
Van der Pool et al. [30]	2010	Operated	1	105/167	NS (*p* = 0.6)	NS (*p* = 0.3)
Brouquet et al. [31]	2011	Operated	1	47/13	S (*p* = 0.003)	NR
Settmacher et al. [7]	2011	Operated	1	158/224	S (*p* = 0.033)	S (*p* = 0.003)
Swan et al. [32]	2011	Operated	4	577/625	NS (*p* = 0.530)	NR
Furukawa et al. [33]	2012	Unresectable	1	26/14	NS (*p* = 0.085)	NA
Vigano et al. [34]	2012	Operated	1	182/194	NS	NR
Dexiang et al. [35]	2012	All	3	1061/552	S (*p* < 0.001)	NR
Gur et al. [36]	2013	Operated	1	79/79	NS (*p* = 0.14)	S (*p* = 0.04)
Nanji et al. [37]	2013	Operated	1	125/195	S (*p* = 0.003)	NS (*p* = 0.092)
Ribeiro et al. [38]	2012	Operated	3	89/81	NS (*p* = 0.162)	NS (*p* = 0.214)
John et al. [39]	2013	Operated	4	257/174	NS (*p* = 0.253)	NR
Hackl et al. [3]	2014	All	2	1019/407	NS (*p* = 0.799)	NR
Kumar et al. [40]	2014	All	1	1054/542	S (*p* = 0.003)	NR
Kuo et al. [41]	2015	Operated	2	104/55	S (*p* = 0.001)	NR
Ali et al. [42]	2015	Operated	3	66/50	NS (*p* = 0.997)	NR
Kawamura et al. [43]	2016	Operated	1	34/38	S (*p* = 0.010	NS
Lemke et al. [44]	2016	Operated	3	68/84	S (*p* = NR)	NR
Miller et al. [45]	2017	Operated	3	181/46	NS (*p* = 0.58)	NS (*p* = 0.87)
Angelsen et al. [46]	2017	Operated	4	39/488	NS (*p* = 0.068)	NR
Bartolini et al. [47]	2018	Operated	1	39/31	NS (*p* = 0.085)	S (*p* = 0.0001)
Margonis et al. [48]	2018	Operated	3	266/583	S (*p* = 0.02)	NS (*p* = 0.58)
Marques et al. [49]	2018	Operated	3	95/55	NS (*p* = 0.505)	NS (*p* = 0.07)
Memeo et al. [50]	2018	Operated	4	753/1031	NS (*p* = NR)	NR
Quireze et al. [51]	2018	All	3	38/16	S (0.036) metachronous worse	NS (*p* = 0.629)
Suthananthan et al. [52]	2018	All	1	276/98	NS (*p* = 0.172)	NR
Strandberg et al. [53]	2018	Operated	1	146/138	S (*p* = 0.031)	NR
Zhao et al. [54]	2018	Operated	1	172/71	NS (*p* = 0.110)	NS (*p* = 0.088)

*Definition: 1, Time of primary tumour diagnosis/operation; 2, 3 months after primary tumour diagnosis/operation; 3, 6 months after primary tumour diagnosis/operation; 4, 12 months after primary tumour diagnosis/operation. *NA* not applicable, *NR* not reported, *S* significant, *NS* non-significant, *OS* overall survival, *DFS* disease-free survival

**Table 4:** Summary of time points for defining synchronous vs. metachronous and prognostic significance as measured by OS and DFS in publications in 2005–2018

Defining time point	Studies (*n*)	Prognostic value			
		OS significant	OS non-significant	DFS significant	DFS non-significant
Primary tumour diagnosis/surgery	18	9	9	4	8
Three months post-primary tumour diagnosis/surgery	4	2	2	0	1
Six months post-primary tumour diagnosis/surgery	12	4	8	0	5
Twelve months post-primary tumour diagnosis/surgery	7	0	7	0	1
Total	41	15	26	4	15

*OS* overall survival, *DFS* disease-free survival

## Discussion

The present study questions the prognostic impact of the time of liver metastases detection in CRC and also the meaningfulness of time points other than the time of primary tumour diagnosis in non-operated patients and time of primary tumour surgery in operated patients as definition of synchronous detection. In a well-defined cohort from a well-defined geographical region in Sweden, no significant difference was seen in survival as measured from time of detection of liver metastases to death between the two groups, irrespective of time points being used.

In patients with CRCLM, the proportions of tumours being detected synchronously and metachronously and the prognostic value of this distinction have been reported in numerous studies [3, 9]. Reports addressing the issue differ considerably regarding the time points used as cut-off for defining synchronous vs. metachronous detection. Furthermore, there are wide variations in the number of subjects and the proportions of synchronous vs. metachronous detection in the included cohorts. Although a trend was seen in patients with metachronous detection having a better OS with early time points as cut-off (50% of studies showing statistically significant differences using a 0- or 3-month definition) as opposed to later time points (50% for a 6-month and 0% for a 12-month definition), there is no compelling evidence that the distinction has any prognostic value. In a study by Furukawa et al., the prognostic value of synchronous vs. metachronous detection was investigated in a cohort of patients with unresectable liver metastases. With the cut-off defined as the time of primary tumour evaluation, no difference in the OS of patients was found [33]. Adam et al. took a consensus approach to the same question and concluded that metachronous tumours are regarded as having a better biology and better survival, and the conclusion was illustrated with survival curves from LiverMetSurvey [9].

With current trends and developments in CRC and CRCLM treatment, establishing the presence of liver metastases at the time of diagnosis of the primary tumour has important therapeutic implications [6, 55]. Firstly, surgical intervention for the CRCLM needs to be coordinated with the surgery for the primary tumour. Different surgical options, for example the liver first approach or simultaneous resection of primary tumour and liver metastases, need to be considered [10, 56–58]. Secondly, it may also influence the use and timing of oncologic treatment, with neoadjuvant chemotherapy increasingly being favoured in patients with liver metastases detected before surgery of the primary tumour [11–13].

Terminology frequently used in the literature, for example *development* of synchronous vs. metachronous metastases, implies two different clinical entities, creating the unfounded impression that the metastatic events occur during the respective periods. The mechanism for CRC metastases to the liver has been described in detail, with tumour cells entering the liver either via the portal vein or hepatic artery, the common point of entry being the sinusoidal space [59]. Whether the risk for new metastases to the liver ceases at removal of the primary tumour is unclear. Patterns of hepatic recurrence observed in a cohort of patients transplanted for CRCLM suggest that previously undiagnosed lung or lymph node metastases could be the source of liver metastases in the transplanted liver [60, 61]. The development of liver metastases in the transplanted liver in the absence of any other metastases suggests that viable tumour cells may persist in the circulation after elimination of the primary tumour and resection of the whole liver with liver metastases. It is however more plausible that pre-operatively present but undetected hepatic lesions account for the vast majority of liver metastases detected after operation of the primary tumour. These lesions are therefore potentially detectable at initial liver work-up.

In the light of the above-mentioned, we propose a standardized definition for synchronous vs. metachronous detection of CRCLM that will be rational, semantically correct and will have a clear clinical application, namely the time of operation for the primary tumour in operated patients and the time of diagnosis of the primary tumour, including the metastatic work-up, for patients treated non-operatively for the primary tumour as cut-off. The rationale for having the cut-off for synchronicity during and not before the primary tumour operation is that, even though establishing the presence of metastases during the pre-operative work-up of the primary tumour is optimal in terms of treatment planning, intra-operative detection still to some extent offers the possibility of changing the treatment plan if needed. Intra-operative detection of pre-operatively undiagnosed liver metastases is fortunately an increasingly rare event due to high-quality imaging using high-end technology and state-of-the-art protocols during work-up [62]. The proposed definition will also focus attention on the effectiveness of hepatic imaging at the time of detection of the primary tumour, the proportion of metastases detected before operation for the primary tumour being directly related to the quality and diligence of the imaging strategy. This could serve as a robust parameter of pre-operative imaging quality control. A randomized trial has shown that MRI with liver-specific contrast is superior for detection of CRCLM, as compared to contrast-enhanced CT and MRI with extracellular contrast [63]. Health economic studies suggest that an MRI first approach is from a cost perspective comparable to a step-up approach with contrast-enhanced CT first [64, 65].

There are, however, a number of limitations that need to be considered in the interpretation and generalization of results. The SCRCR only includes the Swedish population, a relatively homogeneous group in terms of ethnic diversity, and results may not be generalizable. In a previous publication on the total CRC patient cohort from which the CRCLM patients were included in this study, a Cox regression analysis, including age, sex, tumour factors (tumour stage, nodal stage, right- vs. left-sided), number and size of liver metastases, time of detection (synchronous vs. metachronous), liver resection and the presence of lung metastases, was performed. In the multivariate analysis age, primary tumour origin (midgut vs. hindgut), size of largest liver metastasis and liver resection significantly predicted survival, while synchronous vs. metachronous (HR 0.91, 95% CI 0.64–1.30) did not significantly influence survival [4]. In this article, patients that were operated and patients not operated for CRCLM were assessed separately. Ideally, additional treatment such as neoadjuvant, adjuvant and palliative chemotherapy therapy should have been controlled for in a multivariable analysis, but the rather small CRCLM cohort precludes further subgroup analysis.

Although the PRISMA statement guidelines were adhered for extraction of published data required for the study (definitions for and impact of synchronous vs. metachronous detection on prognosis at the different time points), a formal systematic review was not performed. A systematic review and meta-analysis to assess the prognostic value of synchronous vs. metachronous detection using the proposed definition is highly desirable, as this may clarify the impact on prognosis.

## Conclusion

This study, to our knowledge, is the first to address the issue in a well-validated population-based cohort, and it fails to show any prognostic value in distinguishing synchronous from metachronous detection of CRCLM for any of the previously reported defining cut-off points, neither for operated patients nor for patients treated with palliative intent. We suggest using primary tumour diagnosis/operation as standard cut-off point to define synchronous/metachronous detection as a clinically relevant definition.

## Data Availability

The datasets generated and/or analyzed during the current study are not publicly available since it was not originally stated in the ethical application and subsequent approval.

## Abbreviations

CRC: Colorectal cancer
CRCLM: Colorectal cancer liver metastases
DFS: Disease-free survival
OS: Overall survival
SCRCR: Swedish Colorectal Cancer Registry
